# Knowledge, attitude, and practice of pre-diabetic older people regarding pre-diabetes

**DOI:** 10.1186/s12877-024-04864-y

**Published:** 2024-03-18

**Authors:** Vahid Pakpour, Fatemeh Molayi, Hossein Nemati

**Affiliations:** 1 Department of Community Health Nursing, Member of Geriatric Health Group and Aging Research Institute, Tabriz University of Medical Sciences, Tabriz, Iran; 2 Department of Community Health Nursing, Tabriz University of Medical Sciences, Tabriz, Iran; 3 Department of Community Health Nursing, Student Research Committee, Tabriz University of Medical Sciences, Tabriz, Iran

**Keywords:** Knowledge, Attitude, Practice, Pre-diabetes, Older people

## Abstract

**Background:**

One of the risk factors of diabetes is the pre-diabetes stage which is significantly prevalent in older people. Knowledge, attitude, and practice of the pre-diabetic stage are of great importance and can decrease complications. The present study aimed to determine the knowledge, attitude, and practice of the pre-diabetic older people.

**Methods:**

: This cross-sectional study was conducted from April 2022 to August 2022 on 219 pre-diabetic older people referring to Sina Hospital in Tabriz, one of the most populated cities in the northwest of Iran. Data were collected using questionnaires of Knowledge, Attitude, Practice-Prediabetes Assessment Questionnaire (KAP-PAQ). The data were analyzed by SPSS 21.

**Results:**

The mean scores of knowledge (in the range of 0–17), attitude (in the range of -10, + 10), and practice (in the range of 0–26) were 1.72 ± 1.0, 2.24 ± 1.92, and 5.76 ± 2.61, respectively. The older people’s knowledge and practice levels in the pre-diabetes stage were low and about 50% of them had negative views. According to the Spearman correlation test, there was a positive significant relationship between the older people’s knowledge and practice (*p* < 0.001, *r* = 0.234).

**Conclusions:**

The older people in the pre-diabetes stage had low knowledge and attitude and a negative viewpoint towards correcting lifestyle on diet, exercising and physical activity, weight control, diagnostic and screening methods. Increased knowledge about pre-diabetes and strengthened positive attitude towards correcting lifestyle through counseling as well as empowering the pre-diabetic older people can increase the efficiency of pre-diabetes prevention and control programs and prevent its progression to the diabetes stage.

## Background

The older people population is increasing all over the world and it is predicted to reach 1.3 billion people by 2030 and 2 billion people by 2050. In 2050, 80% of older people will be living in low- and middle-income countries [1]. In addition to social and economic issues, chronic diseases such as diabetes affect older people’s quality of life. One of the risk factors of diabetes is the pre-diabetes stage which is prevalent among older people. The prevalence of pre-diabetes and diabetes significantly increases with age. It is estimated that in the United States about 50% of older people have pre-diabetes factors [2]. In Iran, on average, one out of every three people over 60 years old has diabetes. Therefore, due to the higher prevalence of diabetes in older people, special attention should be paid to this age group [3]. The prevalence of prediabetes in Iranian adults and older people is increasing rapidly. In a national cross-sectional survey that was conducted among 35–70 year olds with different ethnicities between 2014 and 2020, the prevalence of prediabetes was reported as 25.4% [4]. The background of diabetes is the beginning of the pre-diabetes period. This phenomenon can initiate diabetes in patients [5].

In prediabetes, blood sugar levels are higher than normal, but not high enough to be diagnosed as type 2 diabetes [6]. Pre-diabetes can not only lead to diabetes, but it is an independent risk factor for a range of diseases, including endothelial dysfunction, atherosclerosis, and cardiovascular disease, which become more important with age [7]. Furthermore, it has been indicated that pre-diabetes increases the risk of hospitalization and mortality, especially among older people [8].

The pre-diabetes stage in older people is sensitive. The knowledge of older people about their nutrition and lifestyle can be very vital and lead to their improved health [9]. Knowledge about diabetes and pre-diabetes can play an important role in motivating individuals to prevent and minimize the complications of diabetes [10, 11]. The lack of knowledge about prediabetes is associated with patients’ reluctance to change lifestyle behaviors [12]. Therefore, considering individuals’ readiness and willingness to make changes, as well as their knowledge and understanding of prediabetes, is an important starting point for engaging individuals in health promotion programs and prediabetes management [13].

Another dimension that should be considered in the pre-diabetic older people is their attitude which can affect their behavior. Attitude is indeed the individuals’ underlying beliefs and can be influenced by their mental opinions and feelings [14]. Individuals’ beliefs can play an important role in their attitudes toward their lifestyle, habits, and behavior [15]. Inadequate care in diabetes is mostly related to the inappropriate attitude of the personnel and patients, and patients who have a positive attitude are more successful in diabetes control and care [16]. Pre-diabetes has the potential to progress to diabetes, but it can also be prevented. Hence, it is crucial to understand the attitude towards preventing pre-diabetes and its associated complications, as this knowledge can pave the way for adopting behaviors that promote a healthy lifestyle [17].

Change in individuals’ knowledge and attitude towards a certain subject can change their behavior and attitude towards that subject, which can be significant and considerable [18]. The older people’s practices in pre-diabetes and diabetes periods are very important. Health care will be inefficient unless correct health behaviors are ensured [19]. Improving people’s knowledge can positively influence their attitude on prediabetes and diabetes, leading to a shift in their behaviors towards embracing healthy lifestyles. This may include practices such as calorie control, adequate sleep, and consistent physical activity [20].

Since the prevalence of diabetes is high in older people and the old people are considered to be the vulnerable population against this disease, diabetes can be controlled by timely diagnosis in the early stages and its complications can be delayed. Therefore, it is necessary to identify the patients in the pre-diabetes stage and before diagnosing diabetes, measure their knowledge, attitude, and practice, and provide a training and care program to prevent the complications and consequences of the disease. In a systematic review conducted in 2023 [12] to investigate the knowledge, attitudes, and practices of patients with prediabetes regarding prediabetes, researchers found that a large proportion of patients (93.7%) were unfamiliar with terms such as “prediabetes,” “impaired fasting glucose,” and “impaired glucose tolerance,” or were simply unaware of them. In middle-income countries, around one in three patients had adequate knowledge about the risk factors and consequences of prediabetes, while more than half of the patients from high-income countries were aware of these factors. However, most patients struggled to accurately identify the diagnostic criteria and screening methods for prediabetes. Although more than half of the individuals knew that lifestyle modifications could help manage prediabetes, approximately 43% believed that medication was the only treatment option. On a positive note, the study revealed that patients generally had favorable attitudes toward preventive efforts for diabetes. They welcomed public health measures such as health promotion and education programs aimed at diabetes prevention. Patients expressed willingness to participate in lifestyle management programs, including weight control, healthy eating, and regular physical activity, in order to adopt a healthier lifestyle. Unfortunately, the study also highlighted poor performance levels among patients with prediabetes. Many reported engaging in unhealthy behaviors such as skipping meals, inadequate fiber intake, excessive fat consumption, and indulging in sugary foods. Additionally, poor sleep quality, a sedentary lifestyle, and difficulties in controlling blood sugar levels were common issues reported by patients. In conclusion, this systematic review study revealed deficiencies in knowledge and suboptimal behaviors related to prediabetes management. However, it also identified positive attitudes toward diabetes prevention efforts [12]. However, there are limited studies on older people’s knowledge, attitude, and practice in the pre-diabetes stage, and most studies have focused on the stage after diagnosing diabetes [21–24]. Therefore, the present study aimed to determine the knowledge, attitude, and practice of pre-diabetic older people to provide health planners and policy-makers with valuable information to design interventions for this vulnerable population.

## Methods

This descriptive cross-sectional study was conducted from April 2022 to August 2022 on 219 pre-diabetic older people referring to Sina Hospital in Tabriz, one of the most populated cities in the northwest of Iran. In this study, the sample size after conducting a pilot study on 20 sample patients, taking into account the standard deviation of 0.36 (the largest standard deviation and related to the performance dimension, α = 0.05 and d = 0.05, 199 people were obtained, which was calculated by considering a 10% drop out of 219 people. The inclusion criteria were the older people in the pre-diabetic stage (the diagnosis of prediabetes is made when the fasting plasma glucose (FPG) is 100–125 mg/dl (5.6–6.9 mmol/l; “impaired fasting glucose” (IFG)), plasma glucose concentration is 140–199 mg/dl (7.8–11.1 mmol/l; “impaired glucose tolerance” (IGT)) 2 h after a 75 g oral glucose tolerance test (OGTT), and/or A1c 5.7–6.4% [25]), who were referred to the diabetes clinic and could communicate verbally to answer the questions. They were included in the study using the convenience sampling method. This means that all patients who visited the Diabetes Clinic of Sina Hospital for a check-up and met the inclusion criteria were invited to participate in the study. A brief explanation of the study’s procedure and research objectives was provided to them. Comprehensive information about the reasons, benefits, and confidentiality of the information was also given to them. If they were interested in participating in the study and gave informed consent, the study questionnaires were completed through interviews conducted by the researcher.

After obtaining the required permits from the Deputy of Research and Technology of Tabriz University of Medical Sciences and the ethics committee (under the code of ethics: IR.TBZMED.REC.1401.119), the written informed consent forms were obtained from the selected older people. The data were collected using the knowledge, attitude, and practice pre-diabetes assessment questionnaire (KAP-PAQ).

Knowledge Attitude Practice-Prediabetes Assessment Questionnaire (KAP-PAQ) is a valid questionnaire to measure pre-diabetic people’s knowledge, attitude, and practice. It was developed by Mohsina et al. in 2020 in India [20]. The questionnaire was developed in several stages including conceptualization, designing the questionnaire, reviewing the texts, examining the experts’ opinions, pre-test, and pilot test. Finally, it was validated using validation methods such as form validity, factor analysis, and Cronbach’s alpha. It contains 30 items that measure knowledge, attitude, and practice of the pre-diabetes stage [20].

The knowledge section includes 10 multiple-choice questions about etiology, diagnosis, consequences, and recommendations about pre-diabetes, with only one correct answer. The wrong answer is scored zero. For every correct answer in questions 1, 2, 7, 8, 9, and 10, 1 point is awarded. For every correct answer in questions 4 and 5, 2 points are awarded. For the correct answer to question 3, 3 points, and the correct answer to question 6, 4 points are awarded. The total score of the questionnaire is 17, and the participants’ knowledge level is classified under three levels: less than 10 (weak), 10–13 (medium), and 14–17(good) [20].

The attitude section measures the participants’ attitudes on a 3-option Likert scale (strongly agree, neither agree nor disagree, and strongly disagree). The items of this section are focused on pre-diabetic people’s attitudes towards correcting lifestyle and gaining beliefs and feelings about pre-diabetes. Every positive attitude is awarded 1 point, every negative attitude is scored − 1, and every neutral attitude is scored zero. The total score is 10. The scores less than zero indicate an extremely negative attitude, the scores between 0 and 2 indicate a negative attitude, the scores between 3 and 6 indicate a neutral attitude, 7 and 8 indicate a positive attitude, and scores 9 and 10 indicate an extremely positive attitude. The range of scores is between − 10 and + 10. Higher scores in the attitude section indicate a positive attitude toward diabetes prevention [20].

The practice section also includes 10 multiple choice questions about the daily habits on nutrition, physical activity, sleep pattern, number of meals, and blood tests. The lowest practice was scored zero, the acceptable practice was scored one, and the highest score was scored 2–4. The sum scores and the highest score indicate a healthier lifestyle. In this spectrum, the maximum score is 26; scores less than 6 indicate very weak practice, scores between 7 and 13 indicate weak practice, scores between 14 and 20 indicate good practice and very good practice is indicated by scores higher than 20 [20].

The validity of the questionnaire was examined using face validity and content validity. The content validity index (CVI) and content validity ratio (CVR) indices for the whole questionnaire were 0.86 and 0.84, respectively. The reliability of the questionnaire was calculated by determining the internal consistency (Cronbach’s alpha coefficient). Regarding the pilot data, the Cronbach’s alpha coefficient for the domains of knowledge, attitude, and practice were 0.65, 0.67, and 0.75, respectively, and the Cronbach’s alpha coefficient was obtained as 0.70 for the whole tool.

The descriptive statistical methods (frequency, percentage, mean, and standard deviation), Spearman correlation coefficient, Mann-Whitney and Kruskal-Wallis tests, and SPSS 21 were used to analyze the data.

## Results

The mean age of the participants was 65.44 ± 5.15 in the age range of 60–88 years. 126 people (57.5%) were male and 93 (42.5%) were female. The average score of knowledge was 1.72 ± 1.0 (the maximum score was 17), of attitude was 2.24 ± 1.92 (out of a maximum score of 10), and of practice was 5.76 ± 2.61 (out of a maximum score of 26).

Table 1 shows the distribution of the older people’s knowledge about the pre-diabetes stage. As it shows, the most correct answer was related to the items “suggesting diet control and doing physical activity for the pre-diabetic older people” (95.9%) and “pre-diabetes disorder can lead to type 2 diabetes” (67.1%). The items “if both parents have type 2 diabetes, what is the chance of developing pre-diabetes?“, “What kind of foods should be consumed regularly in pre-diabetes?“, and “how often should people with pre-diabetes exercise?” with 100% wrong answers had the highest frequency of wrong answers.

**Table 1:** Knowledge about pre-diabetes

Knowledge about pre-diabetes		Correct answer	Correct	False
		Total Score: 17 marks	Number (%)	Number (%)
Prediabetes condition can lead to	a. Type 2 diabetes mellitus b. Type 1 diabetes mellitus c. Both d. None of the above	a (1 mark)	147 (67.1)	72 (32.9)
What is the chance of one getting pre-diabetes, if both their parents have type 2 diabetes mellitus?	a. 25–40% b. 10–15% c. More than 50% d. 0%	c (1 mark)	0 (0)	219 (100)
Which is the best method for detecting pre-diabetes conditions?	a. Blood testing b. Urine testing c. Both d. None of the above	a (3 marks)	1 (0.5)	218 (99.5)
What is the fasting blood glucose level (after an overnight fast of 10 h) in pre-diabetes?	a. 140–199 mg/dl b. 100–125 mg/dl c. < 100 mg/dl d. > 200 mg/dl	b (2 marks)	3 (1.4)	216 (98.6)
Average blood glucose for the past 3 months is given by the _____________(type of a blood test)	a. HbA1c Test b. Fasting blood glucose test c. Fructosamine test d. Oral glucose tolerance test	a (2 marks)	1 (0.5)	218 (99.5)
What is the importance of testing insulin levels along with glucose levels in pre-diabetes?	a. To identify insulin tolerance b. To identify insulin resistance c. To identify insulin overdose d. None of the above	b (4 marks)	1 (0.5)	218 (99.5)
Preferred recommendation for pre-diabetes:	a. Diet control and exercise b. Insulin injections c. Dental checkup d. None of the above	a (1 mark)	210 (95.9)	9 (4.1)
The pre-diabetes should take regularly	a. Foods that are high in fat b. High fiber foods c. Soft drinks and energy drinks d. Foods rich in carbohydrate	b (1 mark)	0 (0)	219 (100)
How often pre-diabetes should do exercise?	a. Once a week for at least 30 min b. Once a month for at least one hour c. Most days of the week for at least 30 min d. None of the above	c (1 mark)	0 (0)	219 (100)
How far weight reduction help pre-diabetes condition in obese patient?	a. Will not help b. Slightly help c. Greatly help d. Unsure	c (1 mark)	2 (0.9)	217 (99.1)

Table 2 shows the distribution of the older people’s attitudes toward the pre-diabetes stage towards pre-diabetes. As is seen, the item of “support of family members is important to deal with pre-diabetes” with 90.4% and the item “people with pre-diabetes should be trained about diabetes mellitus” with 90.0% had the highest rate of agreement, and the item “pre-diabetes disorder is not considered by the society” with 82.2% had the highest rate of disagreement.

**Table 2:** Attitude about pre-diabetes

Attitude about pre-diabetes	Strongly agree (1 mark) Number (%)	Neither Agree nor Disagree (0 mark) Number (%)	Strongly disagree (-1 mark) Number (%)
I can do a lot for my pre-diabetes	170 (77.6)	36 (16.5)	13 (5.9)
Prediabetes should keep their blood sugar close to normal	180 (82.2)	30 (13.7)	9 (4.1)
Control of blood sugar is difficult in pre-diabetes	1 (0.5)	57 (26.0)	161 (73.5)
There is not much use in blood sugar control in pre-diabetes because type 2 diabetes mellitus will happen anyway	0 (0)	59 (26.9)	160 (73.1)
Prediabetes happens only to a cursed person	0 (0)	132 (60.3)	87 (39.7)
People with pre-diabetes should be taught about diabetes mellitus	197 (89.9)	10 (4.6)	12 (5.5)
Prediabetes condition is ignored much by the society	0 (0)	39 (17.8)	180 (82.2)
Support from family is important in dealing with pre-diabetes	198 (90.4)	11 (5.0)	10 (4.6)
Prediabetes should be taught about life style modification	191 (87.2)	21 (9.6)	7 (3.2)
I can lead a normal life in spite of pre-diabetes	193 (88.1)	16 (7.3)	10 (4.6)

Table 3 shows the distribution of the older people’s practice in the diabetes stage. As it is seen, the most correct practices among the participants were related to the consumption of sweet drinks with sugar (carbonated drinks and carbonated fruit drinks) with 61.2% and the consumption of fiber-rich foods such as oats, whole grains, fruits, or vegetable salad as a substitute for regular meals with 48.9%, and the practice of other cases was not good.

**Table 3:** Practice about pre-diabetes

Practice about prediabetes		Score	Number	Percent
How many hours per week do you perform exercises like cycling, walking, yoga etc.?	3 to 6 h a week	4 marks	60	27.4
	1 to 2 h a week	1 mark	0	0.0
	Less than 1 h a week	0 mark	150	68.5
	None	0 mark	9	4.1
How often you consume sugar sweetened beverages (Soda, Carbonated beverages and Non-carbonated fruit drinks)?	Almost never	4 marks	134	61.2
	1–2 times a week	1 mark	0	0.0
	3 or 4 times a week	0 mark	80	36.5
	5 or more times a week	0 mark	5	2.3
How frequently you substitute fiber rich foods like oats, whole grains, fruits or vegetable salads over normal meals?	Almost never	2 marks	0	0.0
	1–2 times a week	1 mark	0	0.0
	3 or 4 times a week	0 mark	8	3.7
	5 or more times a week	0 mark	211	96.3
How often you sleep less than six hours/ night?	Almost never	2 marks	0	0.0
	1–2 times a week	1 mark	0	0.0
	3 or 4 times a week	0 mark	0	0.0
	5 or more times a week	0 mark	219	100.0
How often you skip meals?	Almost never	2 marks	0	0.0
	1–2 times a week	1 mark	0	0.0
	3 or 4 times a week	0 mark	0	0.0
	5 or more times a week	0 mark	219	100.0
How often you consume high fat foods (Like fried snacks and meat, fast foods, chocolates)?	Almost never	2 marks	0	0.0
	1–2 times a week	1 mark	0	0.0
	3 or 4 times a week	0 mark	0	0.0
	5 or more times a week	0 mark	219	100.0
How often you eat food while watching TV/ using mobile phone/ reading books (distracted eating)?	Almost never	2 marks	0	0.0
	Once a day	1 mark	0	0.0
	Twice a day	0 mark	189	86.3
	Every time	0 mark	30	13.7
How long you spend in front of Computer/ TV in a day?	Almost never	4 marks	0	0.0
	1–3 h a day	1 mark	0	0.0
	4–6 h a day	0 mark	203	92.7
	More than 6 h a day	0 mark	16	7.3
How often you check blood sugar at home/lab?	Weekly or monthly once	2 marks	0	0.0
	Once in 2 or 3 months	1 mark	54	24.7
	Once in 6 months or yearly	0 mark	5	2.3
	Never	0 mark	160	73.0
How often you check cholesterol profile at lab?	One or more times in 2 year	2 marks	0	0.0
	Once in 5 years	1 mark	173	79.0
	Once in 10 years	0 mark	42	19.2
	Never	0 mark	4	1.8

Table 4 shows the older people’s knowledge, attitude, and practice in the pre-diabetes stage and the relationship between the variables. As it shows, the older people’s knowledge and practice were at a weak level and about 50% of the older people had a negative attitude towards pre-diabetes. Also, according to the test of correlation between knowledge and practice in pre-diabetic older people, a significant and positive relationship was found (*p* < 0.001, *r* = 0.234).

**Table 4:** Level and correlation of knowledge, attitude, and practice about pre-diabetes

Variable	Status	Range of score	Number	Percent	95% CI	Knowledge	Attitude	Practice
Knowledge	Good	14–17	218	95.5	97.46–99.92	-	*r* = 0.057 *p* = 0.404	*r* = 0.234 *P* < 0.001
	Average	10–13	1	0.5	0.08–2.54			
	Poor	Below 10	0	0.0				
Attitude	Strong positive	9–10	1	0.5	0.08–2.54	*r* = 0.057 *p* = 0.404	-	*r* = 0.059 *p* = 0.387
	Positive	7–8	0	0.0				
	Neutral	3–6	111	50.7	44.11–57.24			
	Negative	0–2	69	31.5	25.72–37.93			
	Strong negative	less than 0	38	17.3	12.91–22.92			
Practice	Very Good	Above 20	0	0.0		*r* = 0.234 *P* < 0.001	*r* = 0.059 *p* = 0.387	-
	Good	14–20	0	0.0				
	Poor	7–13	87	39.7	33.48–46.33			
	Very poor	Below 6	132	30.3	53.67–66.52			

In Table 5, the differences in the level of awareness among older people with prediabetes who visited Sina Hospital in Tabriz are presented based on individual and social variables. As observed in this table, marital status (*p* = 0.021), educational level (*p* < 0.001), physical activity (*p* = 0.001), family history of diabetes (*p* = 0.003), and body mass index (*p* = 0.037) are significantly associated with Knowledge of pre-diabetes. Marital status (*p* = 0.042) is also significantly associated with attitudes toward pre-diabetes and a history of tobacco use (*p* = 0.026) shows a significant relationship with practice.

**Table 5:** Differences in knowledge, attitude, and practice of pre-diabetic older people based on individual and social characteristics

Categorical variables	Status	Knowledge		Attitude		Practice	
		Mean (SD^*^)	P-value	Mean (SD)	P-value	Mean (SD)	P-value
Gender	Male	1.73 (0.58)	0.129	2.32 (1.80)	0.452	5.80 (2.79)	0.764
	Female	1.70 (1.38)		2.12 (2.07)		5.70 (2.34)	
Marital status	Single	1.83 (0.38)	0.021	1.75 (1.86)	0.042	5.25 (2.37)	0.138
	Married	1.77 (1.08)		2.43 (1.82)		5.78 (2.69)	
	Divorced	1.84 (0.80)		2.61 (1.60)		6.84 (1.57)	
	Widow	1.33 (0.71)		1.20 (2.26)		5.43 (2.52)	
Level of education	Illiterate	1.19 (0.69)	< 0.001	1.46 (2.37)	0.171	5.15 (2.32)	0.169
	Elementary	1.61 (0.81)		2.20 (1.82)		5.40 (2.06)	
	Diploma	1.66 (0.51)		2.61 (1.71)		5.84 (2.69)	
	University and higher	2.38 (2.17)		2.07 (1.69)		6.65 (3.39)	
Family income	More income than expenses	2.00 (0.00)	0.457	2.50 (0.70)	0.814	5.50 (3.53)	0.490
	Income equals expenditure	2.00 (0.00)		3.00 (0.00)		4.00 (1.41)	
	Income less than expenses	1.71 (1.01)		2.23 (1.93)		5.78 (2.61)	
Physical activity	Regular	1.90 (0.60)	0.001	2.63 (1.44)	0.104	5.95 (3.11)	0.304
	Occasional	1.77 (1.16)		2.24 (1.94)		5.88 (2.62)	
	Never	1.42 (0.78)		1.90 (2.18)		5.32 (2.03)	
Smoking history	Yes	1.64 (0.55)	0.791	2.61 (2.17)	0.898	5.31 (2.69)	0.026
	No	1.76 (1.18)		2.28 (1.76)		6.02 (2.53)	
Family history of diabetes	Yes	1.37 (1.05)	0.003	1.37 (2.56)	0.135	5.37 (2.81)	0.321
	No	1.76 (0.99)		2.34 (1.80)		5.81 (2.58)	
**Continuous variables**		**Correlation coefficient**	**P-value**	**Correlation coefficient**	**P-value**	**Correlation coefficient**	**P-value**
Age		*r* = -0.126	0.063	*r* = 0.064	0.348	*r* = 0.019	0.779
Body Mass Index (BMI)		*r* = 0.141	0.037	*r* = -0.053	0.438	*r* = -0.057	0.40

*SD (Standard Deviation)

## Discussion

The results of the present study showed that the average score of knowledge about pre-diabetes was 1.72 ± 1.0 (out of 17) and more than 95% of the older people had weak knowledge about diet, exercising and physical activity, weight control, diagnostic and screening methods. Previous studies in Asia and developing countries showed that people with diabetes have insufficient knowledge about diabetes [21, 26–31]. In the study by Nguyen et al., (2020), it was found that the knowledge of people aged above 70 in Vietnam about diabetes was significantly lower than the knowledge of people under 60–69 years old [22]. The researchers in this study pointed out that knowledge is an important factor in preventing pre-diabetes and diabetes. Insufficient knowledge is a risk factor for pre-diabetes and preventive measures, which may lead to a high prevalence of pre-diabetes and diabetes. Prediabetes is a reversible condition that can be prevented from progressing to type 2 diabetes by increasing knowledge about correcting lifestyle [32].

In the present study, about 50% of the older people had a negative attitude towards correcting their lifestyle in the pre-diabetes stage. Borba et al., (2019) showed that more than 85% of the Brazilian older people had a negative attitude toward diabetes [21]. Islam et al., (2014) reported that older people compared to people under 35 years old had significantly negative attitudes toward diabetes [30]. Mainous et al. pointed out that people with negative attitudes towards pre-diabetes were less likely to believe that they can successfully follow the lifestyle changes necessary for preventing diabetes and agree with screening [33]. Therefore, in planning health measures regarding correcting lifestyle in pre-diabetic older people, mental-emotional factors should be considered by interdisciplinary experts.

Furthermore, the results of the study showed that the pre-diabetic older people’s practices of correct consumption of foods, physical activity, sleeping patterns as well as diagnostic and screening methods were not desirable. Ng et al. concluded that appropriate knowledge and attitude factors have led to good measures of disease control [34]. Knowledge can change a person’s thinking process, practices, and attitudes, creating major changes in the person’s behavior. A person’s ability in the light of knowledge appears in the form of appropriate human practices, and in general, a person’s knowledge is the fundamental basis of correct and effective practice in various issues [35].

In the present study, there was a significant and positive relationship between pre-diabetic knowledge and practice. Hyder et al., (2021) also found a positive correlation between knowledge and practice in people with newly diagnosed pre-diabetes. They argued that the more knowledgeable people are about pre-diabetes, they will follow healthier lifestyles to control it [36]. Therefore, an increase in knowledge among older people will improve their practices regarding correct consumption of foods, physical activity, sleeping pattern, and diagnostic and screening methods, which is in turn very important in preventing the progress of pre-diabetes and its control.

Providing healthy lifestyle strategies related to correcting the eating pattern with the aim of weight loss (5–10% of body mass) using real and sustainable dietary approaches with the support of a nutritionist, exercise, and physical activity to facilitate weight control and blood glucose reduction, as well as psychological and social support and timely screening should be considered to increase the results of changing lifestyle in pre-diabetic people [13]. Therefore, increased knowledge and health literacy about pre-diabetes, strengthening positive attitudes towards lifestyle correction through counseling programs, and empowering pre-diabetic older people may increase the efficiency of preventive and control programs for pre-diabetes and diabetes in countries that experience diabetes as a major public health problem.

The results of the present study showed that the Knowledge of older adults about pre-diabetes differs significantly based on body mass index and physical activity. Obesity and sedentary behavior are important risk factors for the progression from pre-diabetes to diabetes [37]. Early intervention in lifestyle, especially weight control and increased physical activity, in individuals with pre-diabetes, can provide an opportunity for promoting health before the irreversible complications of diabetes begin [38]. Therefore, more attention should be given to exercise, dietary habits, and weight control in the development of self-care programs and increasing knowledge among older adults with pre-diabetes.

In the present study, the educational level had a significant statistical difference in terms of knowledge about pre-diabetes. According to Salehi et al., there was a significant relationship between educational level and knowledge about diabetes [39]. The results of the AlSaleh et al. study indicated that individuals with university or higher education had higher knowledge of pre-diabetes [32]. Therefore, the development and implementation of educational programs about pre-diabetes in individuals with lower educational levels can compensate for the lack of pre-diabetes knowledge.

Marital status also had a significant relationship with knowledge and attitudes toward pre-diabetes. Married individuals had more knowledge and a more positive attitude towards lifestyle modification in the pre-diabetes stage. According to Obirikorang et al. study conducted in Ghana in 2016, there was a significant association between marital status and the level of knowledge among patients with diabetes, where married men and women had higher knowledge compared to single individuals [40]. This difference between married and single individuals may be attributed to the support system provided by family and spouses in married individuals.

Furthermore, the results of the present study showed that the practice of older adults with pre-diabetes differs significantly based on a history of tobacco use. Older adults with a history of tobacco use had lower scores in terms of healthy eating patterns, physical activity, sleep patterns, and diagnostic and screening methods in the pre-diabetes stage. In Solberg et al. study, it was found that smokers had less physical activity and fewer visits for diabetes care, A1c testing, foot examination, eye examination, and dental examinations compared to non-smokers. Smokers also reported less inclination towards diabetes self-management activities [41]. Paying attention to the main modifiable risk factor of tobacco use is essential to prevent the onset of diabetes in the pre-diabetes stage, and quitting smoking has clear benefits in terms of reducing the incidence of diabetes [42]. Existing evidence shows that smoking cessation is important for preventing vascular complications in diabetes and may also facilitate blood glucose management [43]. Therefore, smoking cessation programs can be effective in enhancing the practice of older adults in the pre-diabetes stage and preventing the onset of diabetes.

One of the limitations of the present study was that it was performed in one of the hospitals in Tabriz and since social, economic, and cultural factors can affect older people’s knowledge, attitude, and practice, it is suggested to conduct a cross-cultural study in different social and cultural conditions. Another limitation was the use of the self-reporting method, which can lead to boredom, and intentional and unintentional biases in older people, leading to an estimation lower than the reality. However, to reduce the possibility of mistakes or boredom in answering the questions and validating the collected data, it was tried to complete the questionnaires at the most appropriate time so that the accuracy of answers can increase as much as possible. According to the results of the study that older people’s knowledge, attitude, and practice of pre-diabetes were low, it is suggested to conduct an interventional study to promote their knowledge, attitude, and practice of pre-diabetes, and evaluate their status after the intervention.

## Conclusion

The older people had insufficient knowledge and weak practice and a negative attitude towards lifestyle correction regarding correct diet patterns, physical activity, weight control, and diagnostic and screening methods. Increase of knowledge in older people will improve their practices regarding correct consumption of foods, physical activity, sleeping pattern, and diagnostic and screening methods. Increased knowledge and health literacy about pre-diabetes, strengthening positive attitudes towards lifestyle correction through counseling programs, and empowering older people may increase the efficiency of preventive and control programs for pre-diabetes and prevent its progress to the diabetes stage.

## Data Availability

The datasets used and/or analyzed during the current study are available from the corresponding author upon reasonable request.
